# Media Consumption and COVID-19–Related Precautionary Behaviors During the Early Pandemic: Survey Study of Older Adults

**DOI:** 10.2196/46230

**Published:** 2023-05-22

**Authors:** Emily J Smail, Torie Livingston, Adam Wolach, Erta Cenko, Christopher N Kaufmann, Todd M Manini

**Affiliations:** 1 Department of Health Outcomes and Biomedical Informatics College of Medicine University of Florida Gainesville, FL United States; 2 Institute on Aging University of Florida Gainesville, FL United States; 3 Department of Epidemiology College of Public Health and Health Professions University of Florida Gainesville, FL United States

**Keywords:** health communication, COVID-19, older adult, precautionary behavior change, behavior change, behavior, precaution, awareness, behavior modification, media, news, association

## Abstract

**Background:**

During the COVID-19 pandemic, media sources dedicated significant time and resources to improve knowledge of COVID-19 precautionary behaviors (eg, wearing a mask). Many older adults report using the television, radio, print newspapers, or web-based sources to get information on political news, yet little is known about whether consuming news in the early phase of the pandemic led to behavior change, particularly in older adults.

**Objective:**

The goals of this study were to determine (1) whether dosage of news consumption on the COVID-19 pandemic was associated with COVID-19 precautionary behaviors; (2) whether being an ever-user of social media was associated with engagement in COVID-19 precautionary behaviors; and (3) among social media users, whether change in social media use during the early stages of the pandemic was associated with engagement in COVID-19 precautionary behaviors.

**Methods:**

Data were obtained from a University of Florida–administered study conducted in May and June of 2020. Linear regression models were used to assess the association between traditional news and social media use on COVID-19 precautionary behaviors (eg, mask wearing, hand washing, and social distancing behaviors). Analyses were adjusted for demographic characteristics, including age, sex, marital status, and education level.

**Results:**

In a sample of 1082 older adults (mean age 73, IQR 68-78 years; 615/1082, 56.8% female), reporting 0 and <1 hour per day of media consumption, relative to >3 hours per day, was associated with lower engagement in COVID-19 precautionary behaviors in models adjusted for demographic characteristics (β=–2.00; *P*<.001 and β=–.41; *P*=.01, respectively). In addition, increasing social media use (relative to unchanged use) was associated with engagement in more COVID-19 precautionary behaviors (β=.70, *P*<.001). No associations were found between being an ever-user of social media and engaging in COVID-19 precautionary behaviors.

**Conclusions:**

The results demonstrated an association between higher media consumption and greater engagement in COVID-19 precautionary behaviors in older adults. These findings suggest that media can be effectively used as a public health tool for communication of prevention strategies and best practices during future health threats, even among populations who are historically less engaged in certain types of media.

## Introduction

Media exposure has proven to be a powerful conduit in the dissemination of health information and an effective agent in behavior change [1]. During the first few months of the COVID-19 pandemic, it served as a primary source of information to prevent infection and spread of the virus. Precautionary behaviors learned from television, radio, news, and social media acted as a first line of defense against a virus without treatment. Although public health campaigns and messaging through the media have demonstrated small effects on attitudes, beliefs, and behaviors, these effects are believed to accumulate—through reprise and broad reach—to have a large impact on society [2]. During the pandemic, a substantial amount of media content was dedicated to precautionary behaviors (eg, mask wearing and social distancing), yet it is unclear whether the amount of content consumed was sufficient to influence behavior.

Moreover, each type of media (eg, traditional TV news versus social media) could be differentially effective in promoting adoption of precautionary behaviors [3]. For example, traditional TV news is geared toward providing up-to-date information on political and social topics, whereas social media has historically been used to engage with social connections. Thus, traditional news may be more saturated with COVID-19–related information, particularly in terms of behavioral recommendations [4]. Second, whereas journalists have an obligation to present accurate information, social media users tend to have fewer repercussions for spreading falsehoods [5]. Still, explaining this variability in media’s effectiveness for promoting precautionary behaviors is complex, as it reflects a combination of content, medium, repetition, amount of consumption, and population belief.

Older adults, who often have preexisting chronic health conditions, are at the highest risk of severe consequences from COVID-19 infection leading to higher rates of hospitalization and death. Consequentially, this population could largely benefit from the influence of appropriate pandemic-related public health messaging in the media. The percentage of older adults who use social media quadrupled from 11% to 45% between 2010 and 2021 [6]. Despite this, older adults are often underrepresented in research on health messaging, particularly during the pandemic. Distribution of existing surveys are typically performed online, on younger-aged groups (<30 years), or capped at 65 years of age, which limits the generalizability of the findings. Additionally, the depiction of older adults in the media as frail, vulnerable, and helpless contributes to ageism and an age-based health care stereotype that can undermine public health messaging efforts [7-9]. Unregulated social media content has the potential to both fuel and ameliorate this phenomenon. In one example, the idea of COVID-19 as the “BoomerRemover” became popular on twitter, highlighting intergenerational discounting [7,8]. Conversely, media has produced episodes of intergenerational solidarity that fostered a sense of responsibility and togetherness of the young and old [9,10]. The variety of such content masks whether consuming media had a positive, neutral, or damaging impact on practicing pandemic precautionary behaviors.

Our study had three objectives: (1) to assess whether the level of COVID-19–related media consumption was associated with practicing precautionary behaviors, which were intended to reduce infection and spread at the beginning of the pandemic; (2) to evaluate the influence of being a social media user on precautionary behaviors early in the pandemic; and (3) to determine whether change in social media consumption during COVID-19 was associated with engagement in precautionary behaviors. To address the digital divide, we focused on older adults who were targeted through conventional mail to capture individuals who are not regularly on social media or web-based news outlets. Based on the previous literature, we hypothesized that, despite some media-related ageist sentiment, more media consumption, regardless of source, would positively influence precautionary behaviors in older adults.

## Methods

### Data Source

Between May and June 2020, the University of Florida conducted a cross-sectional survey of older adults in the North-Central Florida region to gauge health care behaviors during the COVID-19 pandemic [11]. The timing of the survey release (May 21, 2020) coincided with the Centers for Disease Control and Prevention’s official safety protocols sent on May 20, 2020. At this time, 48,675 cases and 2144 deaths had been recorded in the state of Florida. This survey was posted on the National Institutes of Health Repository of COVID-19 Research Tools under the name “Coping with COVID-19: Impact on technology use, mobility, food security, depression and social isolation.” Respondents took the web-based survey on a voluntary basis, after it was distributed via the University of Florida Research Electronic Data Capture secure system. In addition to social media, email lists, websites, and health articles released by University of Florida Health, a marketing list with addresses of adults ≥60 years of age was purchased to advertise to older adults who are less likely to learn about the study through web-based sources. Direct mail postcards (70,000) that advertised the web-based survey were sent the second week of May 2020. The first response was on May 21, 2020, and the last response was on June 24, 2020.

### Ethical Considerations

The University of Florida institutional review board approved the anonymous web-based survey as exempt from ethics approval. Informed consent was obtained from all participants prior to participation. The data are deidentified, and all results are presented in aggregate form. Participation was voluntary; thus, subjects were not compensated for their responses.

### Media Variables

Respondents were asked 3 questions about media consumption. First, to assess COVID-19–related news consumption, participants were asked, “How many hours per day of media coverage did you watch or listen to about the COVID-19 outbreak?” with answer choices “none,” “some, but no more than one hour per day,” “one to three hours per day,” and “more than three hours per day.” Second, to assess use of social media, participants were asked, “Do you ever use social media sites like Facebook, Twitter, or LinkedIn?” with answer choices “Yes,” “No,” or “Unsure.” Finally, participants reporting social media use were asked, “How has [your use] changed since AFTER the COVID-19 outbreak,” with answer choices “Decreased a lot,” “Decreased a little,” “Stayed the same,” “Increased a little,” and “Increased a lot.” To ensure sufficient sample size for statistical analysis across responses, answers were recategorized into “decreased,” “stayed the same,” and “increased.”

### COVID-19 Precautionary Behaviors

Engagement in COVID-19 precautionary behaviors was measured by asking, “Which of the following have you done since the COVID-19 outbreak to keep yourself safe from coronavirus (in addition or more than you normally do).” Behaviors that aligned with top recommendations (eg, wearing a mask, washing hands, and social distancing) were selected for inclusion. Options included “cancelled a doctor’s appointment,” “washed/sanitized hands,” “cancelled/postponed work activities,” “avoided public places/crowds,” “avoided in-person contact with friends or family,” “worn a face mask,” “worked or studied at home,” “avoided social gatherings,” “avoided in-person contact with high-risk people,” and “cancelled/postponed travel.” Participants could select up to 10 relevant behaviors or select “I am not taking any of these steps.” The behaviors were summed into a behavior score ranging from 0 to 10. Internal consistency of this measure was good (Cronbach α=.70).

### Demographic Characteristics

Covariates included 4 sociodemographic characteristics: age (in years), sex (female or male), marital status (married or not married), and education level (eg, high school or equivalent; post–high school education, any college education, or college degree; and some—or completed—graduate or professional school).

### Statistical Analysis

The analysis was restricted to our convenience sample of 1082 participants with complete information on all analytic variables; 117 (9.8%) were excluded for missing data on at least one analytic variable. We used multivariable, linear regression models to evaluate the association between media consumption and engagement in COVID-19–related protective behaviors, adjusting for sex, age, marital status, and education level. Data structuring and analyses were performed in Stata 17.

## Results

**Figure 1:**
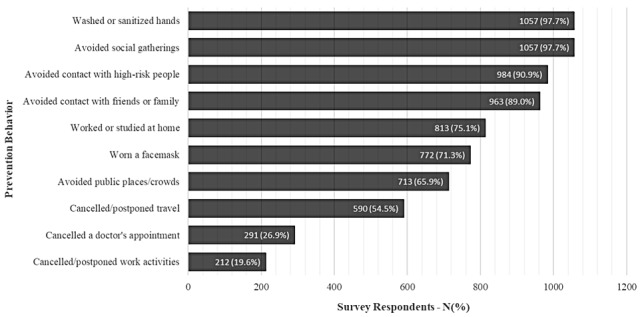
Frequency and percentages of survey respondents endorsing individual COVID-19 prevention behaviors (N=1082).

The mean age of participants was 73 (IQR 68-78) years, and just over half (615/1082, 56.8%) were female. Two-thirds reported they were married. Our sample was highly educated with 44.3% (479/1082) reporting at least some graduate education. Participants reported a median score of 7 (IQR 6-8) out of 10 precautionary behaviors. Of 1082 participants, 33 (3%) respondents reported no media consumption about the pandemic; 420 (38.8%) reported ≤1 hour media consumption per day; 469 (43.3%) reported 1-3 hours media consumption per day; and 160 (14.8%) reported media consumption >3 hours per day. Two-thirds (n=733) reported ever using social media sites (eg, Facebook), and among these respondents, 238 (32.6%) reported increasing their use of social media, whereas 32 (4.4%) reported decreasing use (Table 1). The most common behaviors reported were wearing a mask (n=1057, 97.7%) and washing or sanitizing hands (n=1057, 97.7%), and the least common health behaviors included canceling work activities (n=212, 19.6%) and cancelling a doctor’s appointment (n=291, 26.9%; Figure 1).

Compared to those who reported consuming >3 hours a day of news media, individuals reporting no media consumption and those reporting ≤1 hour a day reported engaging in fewer precautionary measures (β=–2.00, 95% CI –2.67 to –1.33 and β=–.41, 95% CI –0.74 to –0.08, respectively) after controlling for sex, age, marital status, and education (Table 2).

No differences in the number of precautionary behaviors were found between those who did and did not consume social media. Among those reporting social media use, increased use of social media after the COVID-19 outbreak was associated with more precautionary behaviors compared to those reporting no change in use (β=.70, 95% CI 0.42-1.05), whereas decreased use was not associated with engagement in precautionary behaviors (Table 3).

**Table 1:** Sample characteristics.

Variables				Full sample (N=1082)		Social media users (n=730)
**Demographic variables**						
	Age (years), median (IQR)		73.0 (68.0-78.0)		72.0 (68.0-77.0)	
	**Sex, n (%)**					
		Male	467 (43.2)		267 (36.6)	
		Female	615 (56.8)		463 (63.4)	
	**Marital status, n (%)**					
		Not married	395 (36.5)		279 (38.2)	
		Married	687 (63.5)		451 (61.8)	
	**Education level, n (%)**					
		High school or equivalent	137 (12.7)		95 (13)	
		Post–high school or any college	466 (43.1)		318 (43.6)	
		Some graduate or professional	479 (44.3)		317 (43.4)	
**Media variables**						
	**Media consumption, n (%)**					
		None	33 (3)		21 (2.9)	
		<1 hour per day	420 (38.8)		294 (40.3)	
		1-3 hours per day	469 (43.3)		302 (41.4)	
		>3 hours per day	160 (14.8)		113 (15.5)	
	**Social media use, n (%)^a^**					
		Yes	733 (67.7)		—^b^	
		No	349 (32.3)		—	
	**Change in social media use, n (%)**					
		Decreased use	—		32 (4.4)	
		Stayed the same	—		460 (63)	
		Increased use	—		238 (32.6)	
**COVID-19 precautionary behaviors**						
	COVID-19 precautionary behavior score, median (IQR)		7.0 (6.0-8.0)		7.0 (6.0-8.0)	

^a^Three people who reported social media use did not report change in use and were therefore included in the full sample but not among the social media users.

^b^Not applicable.

**Table 2:** Associations between news media and engagement in COVID-19 precautionary behaviors (N=1082).

Characteristics		β (95% CI)
**Media consumption**		
	None	–2.00 (–2.67 to –1.33)^a^
	<1 hour/day	–0.41 (–0.74 to –0.08)^a^
	1-3 hours/day	–0.12 (–0.44 to 0.20)
	>3 hours/day	Reference
**Sex**		
	Male	Reference
	Female	.71 (0.48 to 0.93)^a^
Age		–.02 (–0.04 to –0.00)^a^
**Marital Status**		
	Not married	Reference
	Married	.23 (0.00 to 0.46)^a^
**Education**		
	High school or equivalent	Reference
	Post–high school to college	.30 (–0.04 to 0.65)
	Graduate or professional school	.87 (0.52 to 1.21)^a^

^a^Statistical significance at a *P*<.05 level.

**Table 3:** Associations between social media and engagement in COVID-19 precautionary behaviors.

Characteristics		β (95% CI; N=1082)	β (95% CI; n=730)
**Social media use**			
	No	Reference	Reference
	Yes	.08 (–0.16 to 0.32)	—^a^
**Change in social media use**			
	Stayed the same	Reference	Reference
	Decreased	—	.16 (–0.48 to 0.81)
	Increased	—	.70 (0.42 to 0.99)^b^
**Sex**			
	Male	Reference	Reference
	Female	.71 (0.48 to 0.95)^b^	.77 (0.49 to 1.05)^b^
Age		–.02 (–0.04 to 0.00)	–.01 (–0.03 to 0.01)
**Marital status**			
	Not married	Reference	Reference
	Married	.27 (0.04 to 0.51)^b^	.26 (–0.01 to 0.54)
**Education**			
	High school or equivalent	Reference	Reference
	Post–high school to college	.29 (–0.06 to 0.64)	.40 (–0.01 to 0.81)
	Graduate or professional school	.91 (0.56 to 1.26)^b^	.85 (0.44 to 1.27)^b^

^a^Not applicable.

^b^Statistical significance at a *P*<.05 level.

## Discussion

### Principal Findings

The COVID-19 pandemic highlighted the need to promote health behaviors to limit transmission of the virus. Media consumption (including news and social media) may be an important public health tool to disseminate messages encouraging such behaviors. The purpose of this study was to determine the association between media exposure and precautionary behaviors (eg, hand washing and mask wearing) in older adults living in Florida during the initial stages of the COVID-19 pandemic. Study results showed greater hours consuming news media and increased use of social media post COVID-19 outbreak were associated with higher engagement in precautionary behaviors. Our findings highlight the importance of media as a tool for health information dissemination promoting compliance with policy recommendations aimed at curbing transmission of the disease.

Several mechanisms may explain our results. First, consuming news and social media may increase anxiety about dangers of the virus, subsequently leading to uptake of precautionary behaviors. Early studies following the pandemic outbreak in both national and international settings suggest a mediation effect of anxiety [3,11-14]. For example, Liu et al [15] found that searching for COVID-19 information on digital media was associated with increased uptake of precautionary measures potentially mediated by sense of worry in China. Given COVID-19–related health risks in older adults, this may be a prescient mechanism driving observed relationships. Second, it is possible that consuming social media may lead to social pressures to engage in precautionary behaviors. For example, older adults may see friends and family members wearing masks, and in turn, feel obliged to do the same. Third, media exposure may increase awareness of the pandemic’s public health burden on the population. Specifically, media may highlight the extent that COVID-19 affected the population (eg, case counts and shortage of hospital beds), providing context for individuals outside of immediate experiences in their own communities, and thereby, increasing behavior uptake.

Lastly, due to the cross-sectional nature of this analysis, we cannot rule out the possibility that engaging in prevention behaviors precedes increased media consumption. For example, cancelling activities may result in additional time to consume media. This may be particularly true for social media; during the pandemic, social media was promoted as a potential mechanism for delivering interventions in older adults [16] and as a tool for staying connected [17]. Indeed, older adults used social media as a tool to compensate for losses to in-person social engagement [18]. Therefore, in addition to investigating the mediating role of mental distress and social relationships throughout the pandemic, future research should verify the directionality of the association.

Surprisingly, we did not see differences in reported precautionary behaviors between social media users and nonusers; yet those who increased social media use during the pandemic endorsed slightly more precautionary behaviors on average. One reason for this finding may be that the question asking about use of social media was not specifically geared toward use during COVID-19. Indeed, the question asked about ever using social media; we cannot confirm that participants use social media regularly or that they have used it recently. Thus, the null results may be due to previous users or irregular users of the platforms, who were never exposed to COVID-19 content. Our results may also be impacted by the fact that people who report not using social media are accustomed to receiving news elsewhere (eg, TV) and compensate for the lack of social media time by engaging in alternative news sources. However, in our sample, the two groups report similar amount of COVID-19–related news coverage. Alternatively, it could be that misinformation on social media, which was rampant during the early phases of the pandemic [19], discouraged some users from participation in precautionary behaviors. In fact, a large study by Wong et al [20] in 2021 suggested that social media may have caused more confusion than assistance in promoting certain behaviors [20]. The contradiction between social media as a health promotion tool and a channel for misinformation may counteract and lead to null effects. Additional research is needed to determine what information older adults are exposed to on social media and how this influences participation, or lack thereof, in precautionary behaviors.

Our study’s findings should be interpreted in the context of its limitations. First, our sample may not be representative of all older adults in Florida nor the United States. For example, our sample was predominately White and highly educated. Future research should replicate our findings in more diverse samples. Second, respondents were surveyed 2 months after the pandemic began, and our results may be vulnerable to recall bias, particularly for reports of change in social media use before the outbreak. Additionally, the questions about social media were not specifically geared toward messaging about COVID-19. For example, the second question asked about change in use; we cannot confirm that the participants saw COVID-19–related content on their feed, though we expect that it was ubiquitous during this time. Third, respondents may have been more likely to report certain behaviors even if they did not engage in these measures (ie, social desirability bias). Fourth, we did not assess long-term compliance of engaging in precautionary behaviors; for example, we did not inquire about variability in mask wearing across settings. Finally, we were unable to account for some noteworthy confounders such as political views and trust in the media.

### Conclusions

In summary, we found that increased exposure to media was associated with greater engagement in COVID-19 precautionary behaviors. Our study expanded previous findings by evaluating the association between media and behavior at a critical time in the pandemic (2-3 months after it was declared a national emergency) in a population that is traditionally underrepresented in studies of media messaging. Our findings highlight the significant responsibility of news outlets and social media platforms to disseminate clear and consistent health messages to the public and confirm that this messaging is received and employed by older adults. With the changing course of the pandemic and emergence of new variants, our findings suggest media can be a useful tool for public health communication as the COVID-19 pandemic and future health threats develop.

## Data Availability

Data are available for public use. Requests for data can be submitted to tmanini@ufl.edu.
